# Estimating the Extent of Vaccine-Derived Poliovirus Infection

**DOI:** 10.1371/journal.pone.0003433

**Published:** 2008-10-29

**Authors:** Alison Wringe, Paul E. M. Fine, Roland W. Sutter, Olen M. Kew

**Affiliations:** 1 Department of Epidemiology and Population Health, London School of Hygiene and Tropical Medicine, London, England; 2 Polio Eradication Department, World Health Organization, Geneva, Switzerland; 3 Division of Viral Diseases, Centers for Disease Control and Prevention, Atlanta, Georgia, United States of America; Bill & Melinda Gates Foundation, United States of America

## Abstract

**Background:**

Eight outbreaks of paralytic polio attributable to circulating vaccine-derived poliovirus (cVDPV) have highlighted the risks associated with oral poliovirus vaccine (OPV) use in areas of low vaccination coverage and poor hygiene. As the Polio Eradication Initiative enters its final stages, it is important to consider the extent to which these viruses spread under different conditions, so that appropriate strategies can be devised to prevent or respond to future cVDPV outbreaks.

**Methods and Findings:**

This paper examines epidemiological (temporal, geographic, age, vaccine history, social group, ascertainment), and virological (type, genetic diversity, virulence) parameters in order to infer the numbers of individuals likely to have been infected in each of these cVDPV outbreaks, and in association with single acute flaccid paralysis (AFP) cases attributable to VDPVs. Although only 114 virologically-confirmed paralytic cases were identified in the eight cVDPV outbreaks, it is likely that a minimum of hundreds of thousands, and more likely several million individuals were infected during these events, and that many thousands more have been infected by VDPV lineages within outbreaks which have escaped detection.

**Conclusions:**

Our estimates of the extent of cVDPV circulation suggest widespread transmission in some countries, as might be expected from endemic wild poliovirus transmission in these same settings. These methods for inferring extent of infection will be useful in the context of identifying future surveillance needs, planning for OPV cessation and preparing outbreak response plans.

## Introduction

Much of the success of the Polio Eradication Initiative (PEI) to date can be attributed to massive use of oral polio vaccine (OPV), administered through routine immunisation services and supplementary immunisation activities in the form of National Immunisation Days (NIDs) and subnational immunisation days (sNIDs) in over 100 countries since 1988. Though it is a powerful tool for preventing poliomyelitis, OPV has two disadvantages. In addition to carrying a low risk of vaccine-associated paralytic poliomyelitis (VAPP) among vaccinees or their close contacts, it is now known that vaccine viruses can be serially transmitted through human hosts, and may revert genetically towards wild-type transmissibility and virulence.

The degree of genetic change in vaccine-derived polioviruses (VDPV) is routinely assessed by determining the number of nucleotide substitutions in the VP1 gene, relative to the sequence of the Sabin vaccine viruses, and typically occurs at a rate of approximately 1% per annum[1]–[4]. A virus is defined as a VDPV if it has ≥1% divergence in the VP1 sequence compared to the corresponding Sabin strain. The prefix *c* is used to denote “circulating” (two or more clinical cases), and *i* for VDPV excreted by immunodeficient individuals[5], [6]. The prefix *a* (for “ambiguous”) is used to describe VDPV isolates from persons with no known immunodeficiency or environmental isolates whose ultimate source has not been identified[5].

Eight outbreaks attributable to cVDPV have so far been fully documented: in Hispaniola, Indonesia, Egypt, Madagascar (×2), Philippines, China and Cambodia, resulting in a total of 114 virologically-confirmed (and an unknown number of undiagnosed or unreported) cases[1], [7]–[13]. Two further cVDPV outbreaks were under investigation as this manuscript was submitted, including 5 virologically-confirmed (VC) cases in Myanmar and approximately 130 VC cases in Nigeria and Niger[14]. Single cases of acute flaccid paralysis (AFP) attributable to VDPV have been reported in several other settings[6], [7], [14]–[17] and single VDPV isolates have also been identified in environmental samples and in healthy contacts of AFP cases[7], [14], [18]–[20]

Spread of VDPV infection is likely to be a function of virus transmissibility, population immunity levels, and other population characteristics such as density, socio-economic status, sanitation levels and hygiene-related behaviour, ascertainment efficiency of infection and/or cases, the nature and scope of response activities, and chance. Both the transmissibility and virulence (as measured by the case-to-infection ratio) of VDPVs are difficult to define, as they change over time. These properties take on particular importance as the PEI approaches its final stages.

As part of the plan to discontinue OPV after wild poliovirus (WPV) eradication, there is discussion of vaccine stockpiles, and response strategies to manage any polio outbreaks in the “post-certification” era[21], [22]. Among potential outbreak threats is the possibility that VDPV strains may persist after OPV is discontinued, or as a consequence of the re-introduction of OPV into susceptible populations later, from residual vaccine stores or vaccine manufacturers[23]. Outbreak response strategies should therefore include methods for estimating the likely geographic spread of VDPV infection prior to and during an outbreak, so that vaccination and other response activities cover an appropriate area. Furthermore, stockpiles of OPV, and in particular monovalent OPV (mOPV), have been proposed for future polio outbreak response[22]. The risks associated with such interventions include the potential spread of mOPV-derived VDPV in susceptible populations. Information on the potential spread of VDPV in different contexts will be helpful for developing policies to deal with any such eventualities.

There has been little discussion of the geographic spread of cVDPV outbreak strains, or of the numbers of infections associated with these and other reported VDPV episodes. In this paper, we develop lines of argument to infer the extent of VDPV spread in outbreak populations. We apply the reasoning to data from three of the largest reported cVDPV outbreaks (Hispaniola, Indonesia and Egypt) in order to give crude estimates of the potential numbers of individuals infected with VDPV in each of these settings. We then summarise findings for five smaller cVDPV outbreaks (Madagascar (×2), Philippines, China and Cambodia), and discuss individual VDPV isolates, including iVDPV, and their implications for vaccine-derived virus spread.

## Methods

The data used here come from published reports of eight documented cVDPV outbreaks, shown in table 1. The following lines of argument are used to infer the total numbers infected in each episode:

**Table 1:** Summary of reported cVDPV outbreaks with corresponding estimated number of infections

Country	Number of reported cases	Type	Date of onset of index case	Est. duration of virus circulation	Estimated number of cVDPV infections	References
Hispaniola (pop‡: 16.4 million)	21 VC & 21 PC *	1	July 2000	∼2 years	100,000–200,000	[12], [48], [49]
Madura, Indonesia (pop: 3.5 million)	46 VC and 10 PC	1	June 2005	∼2 years	100,000+	[11]
Egypt (pop: 55.8 million)	30 VC	2	1988	∼10 years	Several million	[1]
Philippines (pop: 75.7 million)	3 VC	1	March 2001	∼3 years	1,000–10,000	[9], [62]
Madagascar (pop: 16 million)	4 VC	2	March 2002	∼2.5 years	10,000–50,000	[10]
China (pop: 1.3 billion)	3 VC	1	May 2004	∼1 year	1,000–10,000	[8]
Madagascar (pop: 16 million)	5VC	2	April 2005	∼1.5 years	10,000–50,000	[13]
Cambodia (pop: 14 million)	2 VC	3	Nov 2005	∼2 years	1,000–10,000	[7], [14], [63]

* VC (virologically-confirmed), PC (polio-compatible)

‡ approximate population size at the time of the outbreak

### 1 Space-time pattern of cases or other isolates

The further apart that cases or (e.g. environmental) isolates are identified in space and time, then the further and longer the implied extent and duration of virus circulation. The total duration of an outbreak, both before and after the initiation of response activities, is also indicative of the extent of infection spread.

Given that virus excretion starts within hours or days after infection, and usually lasts 4–6 weeks[24], the interval between successive infections is likely to average 2–4 weeks (this interval will be inversely related to the population density and the proportion susceptible, since the presence of large numbers of susceptibles will favour transmission early in the course of the infection). This implies an estimate of between 13 and 26 “generations” of transmission per year per virus lineage, at least in immunocompetent populations.

### 2 Population immunity

A low prevalence of immunity to poliovirus infection (i.e. enteric immunity) is likely to encourage cVDPV emergence and spread. The degree of “naturally-induced” immunity can be inferred from the time since the corresponding wild serotype was eliminated, while vaccine-induced immunity can be inferred from the type and extent of past vaccination coverage. Vaccine-induced immunity is complicated however, since not all vaccinated individuals become immune (or seropositive), responses differ by type, and some unvaccinated individuals are immunised by secondary transmission of vaccine virus. Prevalence of immunity to type 2 (at least as reflected by seropositivity) in any age cohort is likely to be equal to or higher than the proportion who have received three routine doses or two doses during campaigns. Immunity (seropositivity) to types 1 and 3 is likely to be lower than the uptake percentages, and to vary considerably between populations, as there is evidence for per dose efficacy as low as 10–20% in some parts of India [25]–[27]. The relationship between vaccination history and seroprevalence will depend upon environment, crowding and hygiene, and deserves further attention. Waning of immunity may need to be considered in future, but is poorly understood.

The maximum number of individuals who could be infected during a cVDPV outbreak is the number susceptible during the period that the virus circulates.

### 3 Age distribution of cases

The age distribution of cases reflects the distribution of immunity in a population at the start of an outbreak, with a higher average age of cases being consistent with a lower crude prevalence of infection immunity, and thus with more extensive (and more rapid) virus spread.

The relationship between the average age of cases (and infected contacts), and the extent of spread is complicated. The case-to-infection ratio of wild poliovirus is age-dependent, with a higher number of infections per case among younger persons[28], [29], which means that the age distribution of disease may not closely mirror the age distribution of infection. Furthermore, although poor hygiene practices conducive to VDPV spread may be most common among young children, older children are likely to travel further, and contact a broader range of individuals, resulting in higher rates of effective contact.

### 4 Outbreak ascertainment and response

The greater the delay between illness onset in the index case, and the initiation of response activities, the more widespread virus circulation is likely to be. Similarly, extensive and high-quality responses are consistent with shorter transmission duration and reduced geographic spread compared to circumstances in which the outbreak responses are of poorer quality.

### 5 Surveillance data

In a polio-free area, the minimum annual expected AFP rate is at least 1 per 100,000 population aged under 15 years. Data on the completeness of AFP reporting and case investigation (including the proportion of AFP cases from whom two adequate stool specimens are obtained) are collected in many countries.

Data on the likely efficiency of AFP case detection prior to and during an outbreak can be used to infer what proportion of cases may have been missed. The extent and results of additional surveillance activities conducted during VDPV circulation, including environmental sampling, community stool surveys, and (retrospective and active) case searches provide information on the extent of VDPV transmission in the community. Extensive sampling with only negative results suggests limited transmission, whereas poor surveillance is at least consistent with widespread infection transmission.

### 6 Social characteristics of cases

The extent of VDPV spread will vary between populations. Cases from disparate social groups suggest wider circulation than those occurring within the same population subgroup. Population mobility will influence the geographic extent of virus spread.

cVDPV isolates identified in crowded populations and/or those with poor hygiene suggest more extensive transmission compared with those found among socially privileged populations. Similarly, populations with a high annual growth rate (particularly where this is determined principally by a high birth rate) will be more prone to extensive VDPV circulation in the absence of high and continuous levels of vaccination coverage, due to the relatively high proportion of new susceptibles being introduced into the population.

### 7 Divergence of VDPV isolates from the reference OPV strain

Polioviruses evolve at an annual rate of approximately 1% of nucleotide substitutions per site in the VP1 gene[1]–[4], [30]–[32]. Thus, sequencing the VP1 region enables a rough estimate of the duration of VDPV circulation. The cumulative number of infections will increase with time and thus the greater the (average) number of nucleotide substitutions of prevalent viruses, the greater the implied extent of (prior) infection transmission.

### 8 Degree of genetic diversity among VDPV isolates

The extent of genetic diversity among isolates provides information on the patterns of poliovirus transmission. In areas with sensitive surveillance, low genetic diversity among contemporary isolates is consistent with narrow chains of transmission with limited branching, whereas high diversity follows early and potentially extensive branching of the chains of transmission. High diversity implies prolonged circulation in populations with large immunity gaps.

### 9 Type-specific properties of VDPVs

OPV viruses and their wild parent strains vary by type in their relative transmissibility and virulence. For OPV, transmissibility and virulence are in the order of 2>1,3[33] and 3>2>1[34] respectively. For WPV, transmissibility is clearly higher than for OPV strains, and probably roughly equal among types, though there is some evidence that type 1 may be more transmissible than types 2 or 3[35] **.** In terms of virulence, the case-to-infection ratios for wild viruses appear to be greatest for type 1 (∼1∶200) and lowest for types 2 and 3 (∼1∶1000)[36], [37]. Attenuation of Sabin strains is based on few nucleotide substitutions for types 2 and 3, with more for type 1[38]–[41]; thus type 2 or 3 Sabin strains may be expected to revert more rapidly than type 1.

### 10 Case-to-infection ratio

The estimated occurrence of 0.36–1.92 cases of VAPP per million initial doses of OPV administered [34], [42]–[46] provides an indication of the virulence of Sabin-derived viruses (immediately or very soon) following vaccine administration. During the course of serial transmission, their virulence is expected to increase by genetic reversion towards 1∶200 (for type 1) or 1∶1000 (for types 2 and 3), although the rate at which this occurs is unknown. The genetic changes associated with more efficient replication in the intestine are also associated with increased neurovirulence, and probably also contribute to more efficient transmissibilities because of higher yields of excreted virus[47]. Thus, given the number of paralytic cases caused by a cVDPV, we can estimate (albeit within a broad range) the number of individuals that may have been infected. The lower the case-to-infection ratio, then the greater the implied extent of infection transmission for a given observed (or inferred) number of cases. This inference also needs to consider that the case-to-infection ratio may vary by age[28], [29].

## Results

### 1 Hispaniola

**Figure 1:**
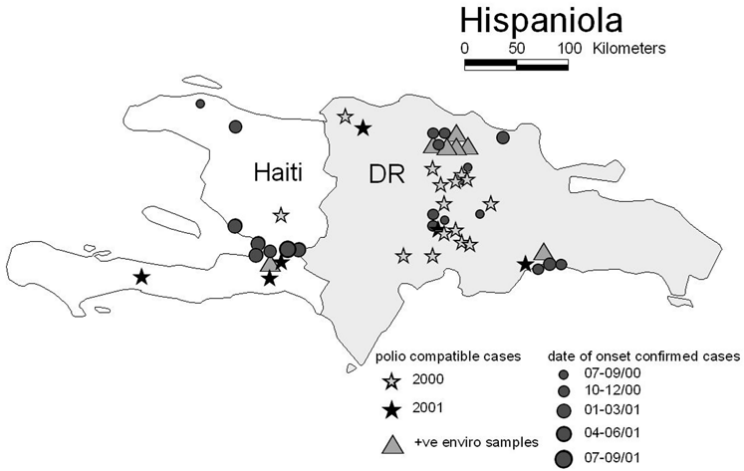
Geographic distribution of virologically-confirmed cases (represented by grey circles) and polio compatible cases (represented by filled stars) associated with type 1 cVDPV outbreak in Hispaniola between July 2000 and July 2001. Environmental samples that were positive for type 1 cVDPV isolates are represented by light grey triangles.

The first recognized cVDPV outbreak occurred on Hispaniola resulting in 21 VC and 21 polio-compatible (PC) cases reported in 2000–1[12], [48] (figure 1).

#### 1.1 Space-time pattern

Although fewer cases were reported in Haiti (8 VC and 4 PC) than in the Dominican Republic (DR) (13 VC and 17 PC), the Haiti cases occurred over a longer duration of time (1 year versus 6 months) suggesting that transmission there was more widespread. Cases were reported from a greater number of departments in the DR than in Haiti (5 versus 3), but this may reflect poor case ascertainment in Haiti, rather than more localized spread of infection.

#### 1.2 Population immunity

The last polio case associated with wild poliovirus was in 1985 in the DR and in 1989 in Haiti, suggesting that “naturally-induced” immunity to infection was restricted to adults over 15 and 11 years of age in the two countries respectively.

NIDs were conducted in DR between 1983 and 1995 and reported routine OPV3 coverage was ∼80% nationally for over a decade prior to the outbreak (although coverage was as low as 20% in outbreak communities). This implies that by 2000, susceptibles were concentrated among the under fives (total ∼1.2 million), of whom approximately (1−0.8)×1,200,000 = 240,000 children <5 years old were unvaccinated when the outbreak occurred. It is likely that at least 200,000 were susceptible to infection.

In Haiti, the prevalence of immunity was probably much lower, with reported national OPV3 coverage fluctuating between 30% and 58% between 1989 and 1999 (but as low as 7% in some communities), and no history of NIDs. This is consistent with up to 70% of children <11 years of age being susceptible to infection when the outbreak occurred, or around 1,600,000 children (0.70×2,300,000).

#### 1.3 The age distribution of cases

The median age of the virologically-confirmed cases was higher in Haiti than in the DR (7 years [range: 2–12 years] versus 3 years [range:9 months–14 years]), reflecting differences in the age distribution of immunity to infection between the two countries. This age difference is consistent with more extensive infection spread in Haiti relative to the DR, both because a greater proportion of the population was susceptible to infection, and also because a broader age range of infected people is likely to correspond to a broader range in contact patterns between infected and susceptible individuals.

#### 1.4 Ascertainment and response

**Figure 2:**
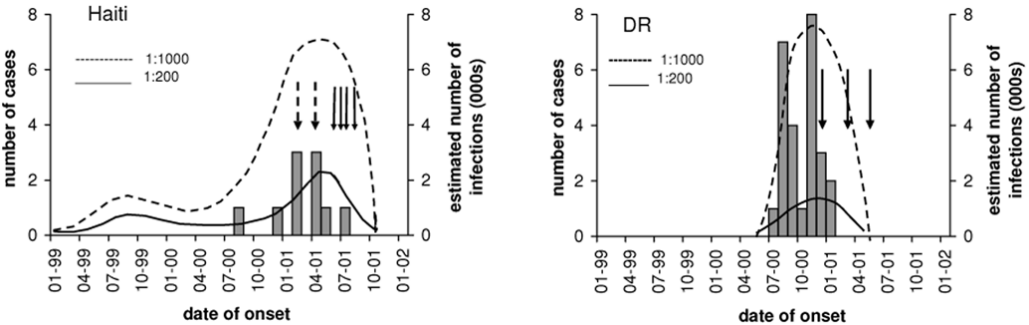
*(adapted from* *Fig 1* *; Kew et al. 2002 Science 296: 356:359)*Temporal distribution of virologically-confirmed and compatible cVDPV cases (grey bars) in Haiti (left hand panel) and in the DR (right hand panel), plotted against the left axis. The estimated number of infections (dotted lines) between the estimated date of the initiating infection and the end of the outbreak is shown against the right hand axis. The number and temporal distribution of these infections represents our best estimate, assuming infection peaks during the summer months, average case ascertainment of 20% in Haiti, and 50% in the DR, and constant case to infection ratios of 1∶200 (----) or 1∶1000 (- - -). Black arrows indicate the dates of NIDs, dashed arrows represent sub-optimal NIDs (in Haiti), and the smaller arrows represent the rolling immunisation campaign in Haiti between May and July 2001.

In the DR, three OPV campaigns were initiated: 5, 7 and 9 months after paralysis onset in the index case (see figure 2), resulting in close to 100% coverage of all children <5 years. The last known case had onset shortly after the first round, suggesting that the response was sufficient to prevent further transmission of the outbreak strain. By contrast, the first two rounds of vaccination at fixed posts in Haiti in early 2001 resulted in less than 40% coverage of the target population, and cases continued to occur. The house-to-house campaign conducted in mid-2001 coincided with the last clinical case and apparent interruption of cVDPV transmission.

#### 1.5 Surveillance data

AFP surveillance performance indicators declined prior to the outbreak. By 1999, the non-polio AFP rate per 100,000 population aged under 15 years was 0.3 in the DR and 0.12 in Haiti, with adequate stool samples taken from only 30% of AFP cases in the DR and from 0% in Haiti. The probability that a non-polio AFP case was reported *and* that adequate stool samples were taken was therefore only around 0.1 in the DR (0.3×0.3) and close to 0 in Haiti. As such, it is highly unlikely that the index cases were the first infected individuals in either country. However, the fact that the virus in the DR index case appears close to ancestral to all subsequent DR viruses is consistent with its being among the earlier infected individuals in that country. No AFP cases were identified retrospectively.

From 12 July 2000 to 31 July 2001, 123 AFP cases were reported from the DR, from which 13 (11%) had type 1 cVDPV isolated. Only 33 AFP cases were investigated during approximately the same period in Haiti, of which 8 (24%) were associated with cVDPV. This corresponds to an annual non-polio AFP rate of 3.8/100,000 in the DR, and ∼0.9/100,000 in Haiti. The difference in the AFP rate in each country and in the proportion attributed to cVDPV is further evidence that more cases went unreported in Haiti than in the DR. The poorer AFP ascertainment in Haiti compared to the DR is consistent with more widespread transmission on this part of the island.

Sampling among healthy contacts of AFP cases was conducted in both countries, with 2/36 contacts positive for type 1 cVDPV in Haiti and 10/205 positive in the DR. Environmental sampling was conducted at the peak of the outbreak from sewage, canals, and public latrines in several sites, with 2/12 (17%) samples from Haiti and 5/43 (11%) from the DR positive for type 1 cVDPV[49]. The positive samples came from two urban sites in the DR and one in Haiti (shown in figure 1), consistent with extensive infection among these populations (where confirmed polio cases also occurred).

#### 1.6 Social characteristics of cases

Up to five million Haitians (55% of the population) live in abject poverty, over four million are without access to safe water and up to three million are slum-dwellers[50]. Worse living conditions in Haiti relative to the DR, combined with lower levels of population immunity mean that VDPV transmission was probably considerably higher than in the DR.

#### 1.7 Divergence of VDPV isolates from the reference OPV strain

Characterisation of isolates from the first two reported cases showed that the outbreak strain had 97.4% and 98.1% sequence identity to Sabin 1, suggesting that the initiating OPV dose occurred in late 1998 or early 1999, 18 months before illness onset in the index case[12]. Assuming an average interval between infections of 2–4 weeks, this implies 20–40 generations of transmission occurred prior to the index case.

#### 1.8 Degree of genetic diversity among VDPV isolates

Sequence analysis of the outbreak isolates suggests that the initiating dose occurred in Haiti, and was followed by repeated divergence of the virus lineages[12]. The virus is estimated to have been introduced into the DR in early 2000, a few months before illness onset in the index case. The VP1 nucleotide diversity of the Haitian isolates was higher than that of the Dominican isolates (0.030 versus 0.007 nucleotide differences per site), consistent with other indicators that transmission was more widespread, and case ascertainment poorer in Haiti than the DR.

#### 1.9 Type-specific properties of the cVDPV

The outbreak strain was derived from Sabin 1, which is the least virulent of the Sabin strains, and is less transmissible than Sabin 2[51]. Though the dynamics of VDPV emergence are poorly understood, the low initial virulence may imply a large number of infections prior to detection of the first cases.

#### 1.10 Case-to-infection ratio

The first two outbreak isolates were shown to be highly neurovirulent in a transgenic mouse model, and replicated to high titres at 39.5°C in cultured cells[12], suggesting that by July 2001, after approximately 18 months of circulation, the case-to-infection ratio was likely to have approached the 1∶200 estimated for wild-type 1 virus.

### Extent of VDPV infection on Hispaniola

VDPV infection appears to have covered a broader area and infected many more people in Haiti than in the DR due to the lower levels of population immunity, poorer sanitation and hygiene, and a slower response to the outbreak on this part of the island. The virological evidence suggests that the virus circulated for over two years in Haiti, and for around nine months in the DR.

In Haiti, response, surveillance and virological data suggest that case ascertainment may have been as low as 20%, which is also consistent with fewer reported cases over a longer time period. A case-to-infection ratio of 1∶200 would suggest that around 12,000 infections (i.e. 12×5×200 = 12 cases×under-reporting factor×case-to-infection ratio), occurred in the outbreak period alone. A case-to-infection ratio of 1∶1000 would imply 60,000 infections. Since the initiating dose was given in Haiti around 20 months prior to the onset of paralysis in the index case, in a population containing over one and a half million susceptibles under 10 years old, it is likely that the total number of infections was at least 100,000.

Thirty polio cases were reported from the DR between July 2000 and January 2001, with surveillance indicators and virological data suggesting that case ascertainment was probably around 50%. If the cVDPV had regained a high degree of virulence (1∶200 case-to-infection ratio) for the entire duration of its circulation, then this would imply that around 12,000 infections occurred during this period. It is likely that the average case-to-infection ratio of the cVDPV was lower than 1∶200, and that case ascertainment was lower than 50% in the early stages of the outbreak (given the results of the retrospective case search and surveillance indicators for 1999). If case ascertainment had been 40% and the average case-to-infection ratio in the order of 1∶1000, then the total number of infections in the DR would have been closer to 75,000.

### 2 Indonesia

In June 2005, a type 1 cVDPV outbreak began in Indonesia, resulting in 46 VC cases[11]. A concurrent polio outbreak associated with wild poliovirus type 1 occurred in Indonesia in the same year.

#### 2.1 Space-time pattern

**Figure 3:**
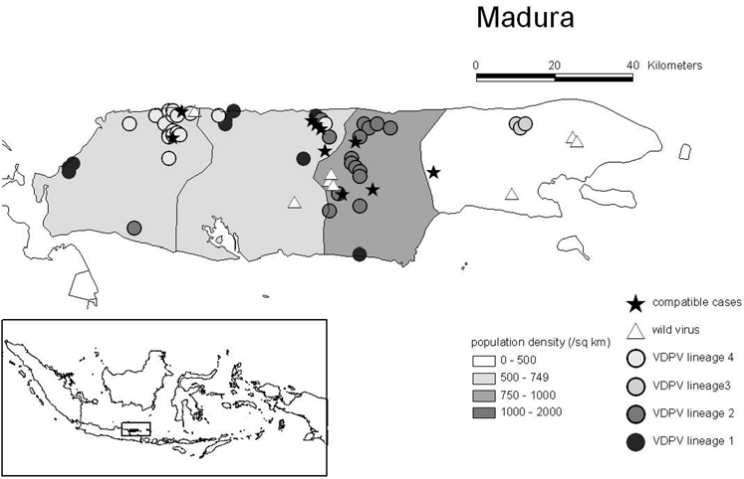
*(adapted from* *Fig 1* *; Estivariz et al. 2008 JID 197: 347–354)* Geographic distribution of virologically-confirmed and polio-compatible type 1 cVDPV cases on Madura, Indonesia between June and October 2005. Population density in Madura for 2005 is shown by district

45/46 virologically-confirmed cVDPV cases occurred between June and October 2005 on Madura island, while one case occurred on the neighbouring island of Java. VDPV cases occurred in all four of Madura's districts (and in 16/68 sub-districts), with several distinct clusters occurring in the rural northern areas of three of the districts (figure 3), indicating extensive geographic spread of the virus. Ten polio-compatible cases occurred during the outbreak period in six sub-districts, with a temporal and geographic distribution compatible with virologically-confirmed VDPV (figure 3).

#### 2.2 Population immunity

Prior to the co-circulating outbreaks of WPV and VDPV in 2005, the last case associated with WPV was reported from Indonesia in 1995, suggesting that naturally-induced immunity was restricted to individuals >10 years old in 2005. NIDs were last conducted in 2001, following which polio immunisation was restricted to routine vaccination. Data on vaccination coverage from convenience samples and non-polio AFP cases suggest that immunisation levels were particularly low in Madura, with over 50% of those <5 years of age having received less than 3 doses of OPV.

With approximately 1.2 million children under 10 years old on Madura island, it is likely that over half a million were susceptible to infection during the period of cVDPV emergence. Within Indonesia, the number of susceptible children under 10 years old is likely to have been over 10 million by 2005.

#### 2.3 The age distribution of cases

The median age of the 45 Madura cVDPV cases was 2 years (range: 6 months to 14 years). 56% (25) of cases were under 3 years old and 20% (9) were 5 years or over. The broad age distribution of the cases is consistent with absence of WPV circulation for over a decade and variable coverage by vaccination services over the same period, giving rise to the potential for extensive geographic cVDPV spread.

#### 2.4 Ascertainment and response

Improved surveillance in Indonesia following the detection of WPV in May 2005 is likely to have accelerated the confirmation of the index case as cVDPV in August 2005, within two months of virus isolation.

**Figure 4:**
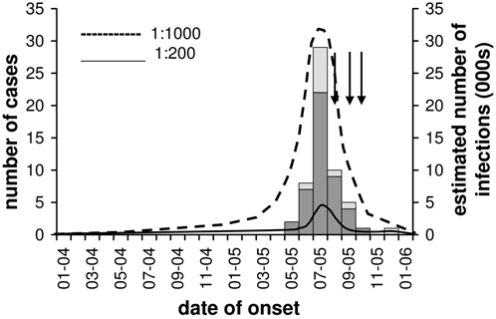
Temporal distribution of virologically-confirmed (grey bars) and compatible cVDPV cases (dashed bars) in Madura, plotted against the left axis. The estimated number of infections (dotted lines) between the estimated date of the initiating infection and the end of the outbreak is shown against the right hand axis. The number and temporal distribution of these infections represents our best estimate, assuming average case ascertainment of 80%, and constant case to infection ratios of 1∶200 (----) or 1∶1000 (- - -). Black arrows indicate the dates of NIDs.

In response to the detection of WPV cases, NIDs were conducted in August, September, and November 2005 (figure 4), with reported coverage >80% in Madura. Illness onset of the last identified cVDPV case occurred in October, while illness onset in the last WPV case on Madura occurred in December.

#### 2.5 Surveillance data

AFP performance surveillance indicators for Madura prior to the recognition of the cVDPV outbreak were low, consistent with poor case identification and the potential for widespread virus circulation.

The non-polio AFP rate per 100,000 children <15 years of age was 0.4 per year in 2003 and 0.7 in 2004. Adequate stool samples were collected from 69% of reported cases in 2004, suggesting that only about half (0.70 * 0.69 = 0.48) of non-polio AFP cases were reported *and* had adequate stool samples collected in the year prior to recognition of the index case.

The non-polio AFP rate increased to 3.3 per 100,000<15 year olds during the first half of 2005 when the cVDPV cases were detected (4/8 reported AFP cases were confirmed as cVDPV during this period), consistent with poor case ascertainment before the recognised outbreak period. AFP surveillance performance was lowest in Sumenep district, which reported the fewest VDPV cases.

Active searches for AFP cases with paralysis onset in the three months prior to the index case were conducted in neighborhoods of confirmed VDPV or WPV cases, and resulted in no further cases being identified.

#### 2.6 Social characteristics of cases

The majority of the Madura cases occurred in sub-districts with a population density of 500–1000 persons/km^2^, and came from poor, rural households, with low utilisation of health services, and suboptimal hygiene and sanitation practices, providing ideal conditions for virus spread.

#### 2.7 Divergence of VDPV isolates from the reference OPV strain

Characterisation of outbreak isolates demonstrated a sequence difference between the outbreak strain and Sabin 1 of 1.1–2.2%, suggesting cVDPV circulation for up to two years prior to detection of the index case, and implying 26–52 “generations” of transmission during this period.

#### 2.8 Degree of genetic diversity among cVDPV isolates

A phylogenetic analysis of the isolates showed four distinct lineages were represented in separate geographic clusters, which were recombinant with other species C enteroviruses. The heterogeneity among the isolates is consistent with extensive transmission of the outbreak strain.

#### 2.9 Type-specific properties of the cVDPV

Type 1 is the least virulent of the Sabin strains, consistent with a large number of infections prior to identification of the index case.

#### 2.10 Case-to-infection ratio

If the outbreak strain had fully regained a case-to-infection ratio consistent with wild type 1 when the cases occurred, and if case ascertainment was complete (46 VC and 10 PC cases), then the minimum number of infected individuals during the outbreak might have been as low as 10,000. A lower case-to-infection ratio of 1∶1,000 would imply in the region of 50,000 to 60,000 infections during the outbreak period.

### Extent of VDPV infection in Indonesia

These arguments indicate that the spread of VDPV infection in the Indonesian outbreak was likely to have been extensive on Madura Island, and limited on Java. Four distinct lineages of the outbreak VDPV circulated for up to two years (26–52 “generations”) before transmission was terminated, and were present in all districts in Madura among subgroups of the population who were poorly immunised, had not been exposed to WPV circulation for over a decade and who lived in conditions conducive to poliovirus spread. Ascertainment is unlikely to have been 100% due to the reported logistical difficulties encountered during the response activities and limited retrospective searches for AFP cases. The apparently later introduction, and relatively limited circulation of type 1 WPV on the same island, also suggests that immunity levels to type 1 may have been recently boosted by the circulating VDPV strain, although vaccination response activities would have limited WPV spread. Taken together, it is not unreasonable to estimate that at least 100,000 individuals were infected in Indonesia during the full period in which the VDPV circulated, with the vast majority residing on Madura.

### 3 Egypt

A retrospective analysis of *stored* poliovirus isolates revealed that type 2 cVDPV circulated in Egypt between 1988 and 1993[1]. Of 30 isolates from this era initially thought to have been wild type 2, all proved to be cVDPVs.

#### 3.1 Space-time pattern

**Figure 5:**
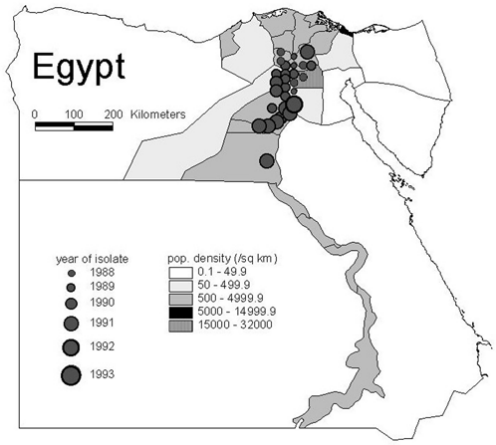
Geographic distribution of virologically-confirmed type 2 cVDPV cases in Egypt between 1988 and 1993. Individual cases are represented by filled circles of different sizes to show the year of paralysis onset. Population density for Egypt in 1990 is shown by governorate.

The recognised cVDPV cases were from 7 of the 27 governorates of Egypt, with onset dates between 1988 and 1993 (Figure 5). The predominance in the north reflects the geographic bias in the collection history of the stored samples. The 7 governorates contain around 50% of the total population.

#### 3.2 Population immunity

Though the last reported wild type 2 polio case in Egypt was in 1979, the exact date of eradication of this serotype is unknown since AFP surveillance began only in 1990[52]. Wild poliovirus types 1 and 3 circulated in Egypt throughout the 1990s, with type 1 still present up to 2005 [53]. National vaccination data for the period are incomplete, but suggest that OPV3 coverage was low but gradually increasing throughout the 1980s.

There were approximately 20 million children under 15, and around 1.25 million births per year, in Egypt throughout the 1980s[54] suggesting that millions of individuals were susceptible to WPV2 infection during the outbreak period.

#### 3.3 Age distribution of cases

Age information is no longer available on the cases from whom the isolates were obtained.

#### 3.4 Ascertainment and response

There was no specific response to the outbreak in Egypt as it was ascertained retrospectively. Transmission is likely to have ceased in the mid-1990s following an increase in OPV coverage[55].

#### 3.5 Surveillance data

The 30 confirmed cases are likely to represent only a fraction of those attributable to the cVDPV as polio surveillance is known to have been poor during the outbreak period. There was no routine AFP surveillance. The stored isolates represent cases visiting particular health facilities after 1988.

#### 3.6 Social characteristics of cases

Data on the social characteristics of the cases have not been reported, but both the living and sanitary conditions, and the population density (among the highest in the world) and frequent mixing patterns in Egypt favour poliovirus spread, as shown by the persistence of wild type 1 until early 2005.

#### 3.7 Genetic divergence of VDPV isolates from the reference OPV strain

**Figure 6:**
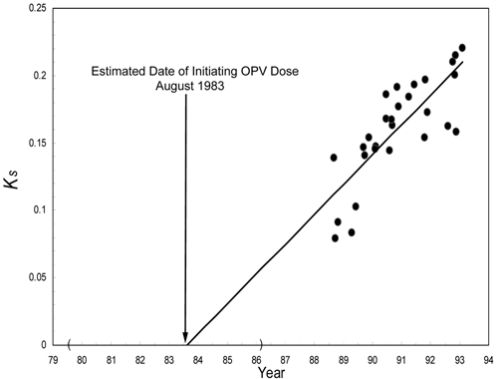
(*From:* Yang et al, *J Virol* 2003; 77:8366–8377) Estimate of the date of initiating OPV dose from the rate of accumulation of synonymous substitutions into VP1 among the 28 type 2 cVDPV isolates for which the dates of sample collection are known. Abscissa: date of sample collection for each isolate. Ordinate: *Ks*, the number of substitutions (Sabin 2 sequence set to zero substitutions) at synonymous sites in VP1. The evolution rate was estimated by weighted linear regression. The 95% CI for the estimated date of the initiating OPV dose, July 1979 to March 1986, is bounded by parentheses along the abscissa.

The outbreak isolates had between 93% and 97% nucleotide sequence identity to the Sabin type 2 strain, and a regression analysis of the VP1 evolution rate suggests that the lineages resulted from an OPV infection that occurred in about 1983 (95% CI: 07/79–03/86), as shown in figure 6. This suggests that cVDPV circulated for approximately 5 years prior to the date of onset of the first stored isolate, and for around 10 years in total.

#### 3.8 Degree of genetic diversity among VDPV isolates

Sequence analyses show a high degree of nucleotide diversity between the outbreak isolates, consistent with multiple, extensive chains of transmission and incomplete case ascertainment[1].

#### 3.9 Type-specific properties of the cVDPV

The Egypt outbreak was associated with type 2 which is the most transmissible of the Sabin strains[34], [51].

#### 3.10 Case-to-infection ratio

**Figure 7:**
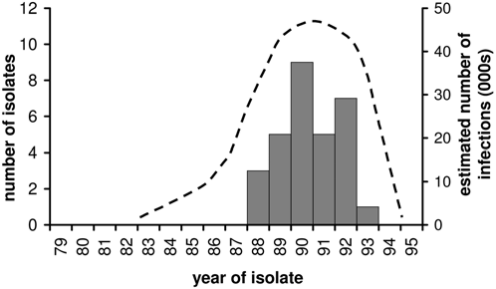
The distribution of reported cVDPV cases in Egypt (grey bars) is shown on the left hand axis. The estimated number of infections (dotted line) between 1983 (when the initiating infection is estimated to have occurred) and the end of the outbreak is plotted against the right hand axis, and represents a best estimate of the distribution of infections. The approximation for the number of infections is represented by the area under the curve, and assumes a constant case to infection ratio of 1∶1000, and average case ascertainment of ∼10%.

The neurovirulence of two isolates from the outbreak was measured in transgenic mice and found to be similar to the prototype wild type 2 strain[1]. Assuming a case-to-infection ratio for wild type 2 of 1∶1,000, the 30 reported cases alone are likely to be associated with around 30,000 infections (figure 7).

### Extent of infection in Egypt

cVDPV infection appears to have been much more extensive in Egypt than in the other known cVDPV outbreaks, with continued circulation for approximately 10 years, the occurrence of recognised cases over a broad area that comprised at least 50% of the total population, and evidence of multiple chains of transmission. Very high population density, particularly in slum areas in “mega-cities” and a high birth rate are likely to have contributed to extensive spread of infection. Virological evidence, along with poor case reporting before the 1990s, suggest that many hundreds of cases were probably associated with the outbreak. Taken together, the evidence indicates that the cVDPV strains became endemic in the population completely filling the niche vacated by the disappearance of indigenous wild type 2 viruses. It is likely that an appreciable proportion (up to several millions) of the approximately 12 million children born in Egypt 1983–1993 were infected with these type 2 cVDPV viruses.

### Smaller cVDPV outbreaks

In addition to these 3 large cVDPV outbreaks, 5 further outbreaks of cVDPV have been documented. Table 2 summarises the findings from applying the same lines of argument to each of these five settings and shows that between 1,000 and 50,000 individuals were likely to have been infected in each outbreak. The virological evidence suggests that the extent of infection was likely to have been particularly limited in the Philippines, with only a few susceptible pockets in the population likely to have been affected. In contrast, the data from Madagascar indicate that conditions were ideal for cVDPV emergence and spread, as confirmed by the occurrence of two distinct cVDPV strains and a single AFP case attributable to VDPV[10], [13].

**Table 2:** Summary of estimated number of cVDPV infections associated with five small-scale cVDPV outbreaks

	PHILIPPINES (type 1)	MADAGASCAR (type 2)	CHINA (type 1)	MADAGASCAR (type 2/3)	CAMBODIA (type 3)
Space-time pattern	3 cases & 1 infected contact between 03–09/2001 on 2 islands 800 km apart; ferry link	4 cases between 03–04/2002 in 3 rural villages in the south-east of the country.	3 cases & 4 infected contacts between 05–08/2004 in 2 villages 40 km apart	5 cases & 12 infected contacts between 04–08/2005 in 5 districts of a rural province in SW of the country	2 VC cases in the capital in 11/05 & 01/06
Population immunity	WPV eradicated in 1993; Sub-optimal NIDS since 2000 Up to 2 million susceptible <5 yrs	WPV eradication in 1997; Last NIDs in 1999 with coverage >90%; Routine OPV3 coverage <40% in 2001; ∼1 million susceptible < 5 yrs in 2001	WPV eradication in 1994. Vaccination coverage <70% in affected villages since 1995. Up to 3.5 million susceptible <5 yrs in affected province (∼1,000 in affected villages)	WPV eradication in 1997; Prior type 2 cVDPV in 2002; Despite SIAs in 2002, routine OPV3 coverage in affected areas <50%; ∼0.8 million susceptible <5 y (mostly <3y)	WPV eradication in 1997; Routine OPV3 overage 82%; but <50% around cases; ∼1 million susceptible <5 yrs
Age distribution of cases	3 cases and infected contact were 8, 3, 1 and 3 yrs old	4 cases aged 6, 9, 14 and 20 mths old	Cases aged 0.9–3.2 yrs; contacts aged 1.4–7.3 yrs	Cases aged 2–3 yrs old; contacts 0.7–6 yrs old	Cases aged ∼18 mths
Ascertainment and response	Index case confirmed cVDPV within 3 mths of virus isolation; 3 OPV rounds, beginning Dec 2001; reported coverage >95%; no further isolations	OPV rounds in affected areas after 8 mths & NIDs after 11 mths; ∼0.5 million children (15% of target) missed NIDs;	Province-wide OPV campaign for <5 yr olds in 08/04; no further isolations of the outbreak strain	2 rounds of SIAs in Sept and Oct 2005; no further cases reported	3 OPV rounds in March-May 06 in high-risk areas; no further virus isolations
Surveillance	Annual non-polio AFP rates in decline since 2001; substantial variation within districts in collection rates for adequate stool specimens.	Poor AFP surveillance indicators in year prior to outbreak (∼1/4 non-polio AFP cases reported *&* had adequate stool samples collected).	Very poor AFP surveillance in affected province in 2004 (<0.1 non-polio AFP/100,000 pop <15);	Poor AFP surveillance indicators in the affected province during the period leading up to the outbreak	Non-polio AFP rates in Phnom Penh and nationwide ∼2/100,000 population <15 yrs prior to the reported cVDPV cases
Social characteristics of cases	Index case from slum area; 2^nd^ & 3^rd^ cases related	Cases related & had history of recent contact	Low population density, very poor & remote villages	4/5 cases in poor, rural villages; 1 case with recent travel history	Cases occurred in very poor communities in the capital
deDivergence of VDPV isolates from the reference OPV strain	3% VP1 sequence difference from Sabin 1: circulating for ∼3 yrs.	∼2.5% VP1 sequence difference from Sabin2: circulation for ∼2.5 yrs.	1–1.2% VP1 sequence difference from Sabin1: circulation for ∼1 yr.	1.1–1.8 % VP1 sequence difference from Sabin2: circulation for ∼1.5 yrs.	1.9–2.4 % VP1 sequence difference from Sabin 2; circulation for ∼2 yrs.
Degree of genetic diversity among cVDPV isolates	Isolates formed single cluster, with high degree of sequence similarity; consistent with narrow chain of transmission.	Isolates formed 2 sub-groups, but > 99% sequence homology in VP1; consistent with narrow chain of transmission.	naIsolates diverged from common ancestor ∼5–6 months before detection of outbreak	3×type 2 lineages with multiple transmission chains & 1 type 3 lineage along a unique and independent chain	The two isolates shared only ∼50% of substitutions, consistent with ∼8 months of independent circulation
Type-specific properties	Type 1 least virulent of Sabin strains, consistent with large number of infections prior to identification of index case.	Sabin 2 most transmissible; reversion towards wild phenotype may be more rapid than for Sabin 1or 3	Type 1 least virulent of Sabin strains, consistent with large number of infections prior to identification of index case.	2^nd^ type 2 cVDPV outbreak in Madagascar may suggest ideal conditions for emergence and spread of cVDPV	Type 3 most virulent of Sabin strains; consistent with fewer infections prior to identification of index case
Case-to-infection ratio	2 case isolates had same virulence as WPV1 when tested in transgenic mouse model; likely to be 600–3000 infections associated with reported cases	If the outbreak strain had WT virulence, then ∼1000 infections per case (though the clustering of cases suggests some predisposing factor)	2 cases associated with a minimum of 400 infections (if outbreak strain had fully regained WT virulence)	Likely to be 1,000–5,000 infections/case. Full case ascertainment would suggest a max of 15,000 infections during outbreak period alone.	If the outbreak strain had WT virulence, would imply a min of 3×200 infections during the outbreak period.
**EXTENT OF INFECTION**	**Likely to be in the range of 1,000–10,000 infections**	**Likely to be in the range of 10,000–50,000 infections**	**Likely to be in the range of 1,000–10,000 infections**	**Likely to be in the range of 10,000–50,000 infections**	**Likely to be in the range of 1,000–10,000 infections/emph>**

### Single VDPV isolate episodes

In addition to these multi-case outbreaks, there have been at least 35 isolations of VDPV strains which appear, because of VP1 divergence ranging from around 1–3%, to have circulated in populations for periods of ∼1–3 years [5]–[7], [14]–[20], [56], [57]. These isolates include 10 derived from type1, 19 from type 2 and 6 from type 3, and have been identified in more than 20 countries over the years 1966 to 2007. Although it is impossible to know how many individuals may have been infected with any of these VDPV lineages, 100 is a minimum, but 1000 a more likely low-end estimate, given their estimated duration of circulation and the estimated annual number of generations of transmission.

### iVDPVs

The long-term excretion of VDPV by some immunodeficient individuals (iVDPV) has been recognized since 1962[58]. Characterization of some of these isolates suggest that, like cVDPV, the annual rate of nucleotide substitution in VP1 is around 1%[30], [31], with chronic infection being observed for over 10 years in some cases[59], [60]. To date, 40 individuals with prolonged iVDPV infection have been identified[7], [14], [56] and (probable) iVDPV isolates from environmental sampling have been identified in several settings[7], [18]–[20]. So far, most cases and isolates have been identified in high- or middle-income communities, where high levels of immunization and good hygiene and sanitation standards serve to minimize the risk of spread. However, there has been one documented episode of iVDPV spread in the US, among the Amish population who often refuse OPV, with 5 infections including an immunodeficient child with long-term VDPV excretion[61].

## Discussion

Person-to-person transmission of VDPV infection for prolonged periods has been recognised in several settings, and has resulted in eight fully documented multi-case outbreaks of polio. In addition, over forty VDPVs have been isolated from individual AFP cases, healthy individuals, immunodeficient individuals and in environmental samples in other settings. The important risk factors for cVDPV emergence-low prevalence of immunity to poliovirus infection, low OPV coverage and poor sanitation-are likely to exist in populations where case ascertainment is incomplete. It is thus likely that the number of cVDPV cases recognised in these eight outbreaks falls well below the true number of cases, particularly in Egypt and Haiti, with the actual incidence of VDPV-attributable paralytic disease likely to have numbered in the many hundreds.

The spread of VDPV infection was most extensive in Egypt, where several million individuals may have been infected by type 2 cVDPV which became endemic following the disappearance, of indigenous wild type 2 virus. In Hispaniola and Indonesia, at least 100,000 individuals are likely to have been infected in each setting, while up to 10,000 infections may have occurred in each of the five smaller outbreaks. The isolates from single AFP cases, healthy contacts and environmental samples identified in other episodes, are together likely to correspond to several thousand infections. A large type 2 VDPV outbreak is currently ongoing, with roughly 130 cases attributed to the independent emergence of seven cVDPV lineages in northern Nigeria [14], where routine trivalent OPV coverage is <30%, and most supplementary immunisation activities have largely used type 1 and 3 monovalent OPV. It is likely that many tens, if not hundreds of thousands of individuals have already been infected with these lineages, but the full impact of this ongoing outbreak remains to be seen.

Each of the eight “outbreaks” ended because of OPV intervention. In Egypt, this intervention occurred as part of the scale-up of routine OPV during the 1990s (and one could question its description as an outbreak, given that the cVDPV had established itself in the population over a period of several years). Special NIDs were organised to control the seven other outbreaks.

Reports of cVPDV outbreaks and references to other VDPV isolates have so far focused on the numbers of clinical cases involved in these episodes. Their full implications come to light when the underlying infection incidence is considered. Insofar as eradication of all poliomyelitis is the PEI target, this will require total cessation of all poliovirus transmission. To describe the problem of vaccine-derived polio as 114 virologically-confirmed cases, worldwide, over some twenty years, gives a very different impression than a description which suggests a minimum of hundreds of thousands, and more likely several million infections by vaccine-derived viruses, some of which became endemic in large populations. It is also possible that other vaccine-derived virus lineages have circulated for limited time periods, but failed to cause any clinical cases and were thus unrecognized[61]. The risk of VDPV appearance and the incidence and spread of these infections will be important considerations for policies relating to the cessation of OPV, for future surveillance needs, and for planning for outbreak control in the future, including stockpiling vaccines.

Although the methods developed here give wide-ranging estimates of the numbers infected during each episode, they nevertheless highlight the need to focus on the extent of infection spread in the event of future VDPV episodes. As such, these methods will become increasingly important for planning surveillance, control and response activities in the current and future stages of the eradication programme, as attention focuses towards reducing cases, and ultimately infections, to zero.
